# Vav1 promotes lung cancer growth by instigating tumor-microenvironment cross-talk via growth factor secretion

**DOI:** 10.18632/oncotarget.2400

**Published:** 2014-08-27

**Authors:** Shulamit Sebban, Marganit Farago, Shiran Rabinovich, Galit Lazer, Yulia Idelchuck, Lena Ilan, Eli Pikarsky, Shulamit Katzav

**Affiliations:** ^1^ Departement of Developmental Biology and Cancer Research, Institute for Medical Research Israel-Canada, Hadassah Medical School - Hebrew University, Jerusalem, Israel; ^2^ Department of Immunology & Cancer Research and Department of Pathology, Institute for Medical Research Israel-Canada, Hadassah Medical School - Hebrew University, Jerusalem, Israel

**Keywords:** Vav1, CSF1, Lung Cancer, Growth Factors

## Abstract

Vav1 is a signal transducer that functions as a scaffold protein and a regulator of cytoskeleton organization in the hematopoietic system, where it is exclusively expressed. Recently, Vav1 was shown to be involved in diverse human cancers, including lung cancer. We demonstrate that lung cancer cells that abnormally express Vav1 secrete growth factors in a Vav1-dependent manner. Transcriptome analysis demonstrated that Vav1 depletion results in a marked reduction in the expression of colony-stimulating-factor-1 (CSF1), a hematopoietic growth factor. The association between Vav1 expression and CSF1 was further supported by signal transduction experiments, supporting involvement of Vav1 in regulating lung cancer secretome. Blocking of ERK phosphorylation, led to a decrease in CSF1 transcription, thus suggesting a role for ERK, a downstream effector of Vav1, in CSF1 expression. CSF1-silenced cells exhibited reduced focus formation, proliferation abilities, and growth in *NOD/SCID* mice. CSF1-silenced H358 cells resulted in significantly smaller tumors, showing increased fibrosis and a decrease in tumor infiltrating macrophages. Finally, immunohistochemical analysis of primary human lung tumors revealed a positive correlation between Vav1 and CSF1 expression, which was associated with tumor grade. Additional results presented herein suggest a potential cross-talk between cancer cells and the microenvironment controlled by CSF1/Vav1 signaling pathways.

## INTRODUCTION

Growth factor signal transduction pathways mediate processes such as survival, proliferation, differentiation, migration and multicellular communication in metazoans [1]. The activity of signal transducers is normally tightly controlled; however, mutation and other genetic alterations can perturb regulation of molecules such as receptor tyrosine kinases (RTKs), cytoplasmic TKs and other signal transducers, resulting in malignant transformation [2].

One signal transducer that has been shown to be deregulated in several human cancers is Vav1. First isolated as an oncogene, Vav1 is a hematopoietic specific GDP/GTP exchange factor (GEF) for Rho/Rac GTPases [3]. Rho/Rac activation leads to cytoskeletal rearrangement inducing functional changes in different cells, including immune cells [4,5]. Increasing evidence demonstrates that Vav1 also modulates several signaling pathways independent of its exchange activities [6,7]. Others and we detected ectopic expression of Vav1 in neuroblastoma [8], pancreatic [9], lung [10] and breast cancers [11]. We demonstrated Vav1 expression in malignant human lung cancer specimens (~44%) and in human lung cancer cell lines (~42%). siRNA-mediated knockdown of Vav1 in human lung cancer cells reduced proliferation in agar and tumor growth in nude mice, despite the presence of mutant K-Ras in these cells, suggesting that Vav1 plays a critical role in lung cancer [10]. These findings suggest that Vav1, when expressed ectopically outside of the hematopoietic system where it normally functions, is stimulated by various growth factors and contributes to signal transduction processes that lead to malignancy [8-11]. Indeed, Vav1 was shown to be tyrosine phosphorylated following EGF stimulation of neuroblastoma, pancreatic and lung cancer cells [8-10]. Similarly, EGF and PDGF stimulate tyrosine phosphorylation of Vav1 when it is ectopically expressed in NIH3T3 cells [12,13]. NGF was also shown to activate Vav1 in hematopoietic cells [14]. Thus, potentially, Vav1 can be tyrosine phosphorylated in cancer cells through numerous tyrosine kinase growth factor receptors (RTKs), partly not analyzed yet.

Following tyrosine phosphorylation, Vav1 might contribute to malignancy by activating signaling cascades through its GEF activity. Indeed, Vav1 regulates cytoskeleton reorganization in response to extracellular stimuli and participates in cancer cell invasion [15]. For instance, a truncated version of Vav1 leads to actin cytoskeleton reorganization and transformation of NIH3T3 fibroblasts [16] and Vav1-transformed NIH3T3 fibroblasts metastasize to the lungs of mice injected intravenously with the transformed cells [17]. Recent studies in pancreatic cancer [9] and lung cancer [10] cells that express Vav1 clearly showed that Vav1 functions as a GEF for Rac1 GTPase following EGF stimulation. Knockdown of Vav1 by RNAi in melanoma cells led to impaired activation of the Jak/Vav1/RhoGTPases (Rac1 and RhoA) pathway, blocking up-regulation of MT1-MMP by CXCL12, a mechanism that contributes to melanoma cell invasion [18]. Furthermore, it was demonstrated that the large GTPase Dynamin 2 potentiates invasive migration of pancreatic tumor cells through stabilization of the Rac1 GEF Vav1 [19]. Also, Vav1 was shown to promote the matrix-degrading processes underlying pancreatic tumor cell migration through its GEF activity [20]. Thus, when expressed ectopically, Vav1 is considered to function as a central regulator and major driver of invasive matrix remodeling by pancreatic tumor cells [19, 20]. Together, the aforementioned findings imply that GEF activity is critical for Vav1′s role in cancer cell migration and invasion and suggest that ectopically expressed Vav1 acts as an upstream activator of Rac1, RhoA and possibly Cdc42 signaling pathways in response to extracellular stimulation, leading to cytoskeleton changes and ultimately to increased cell motility [19, 20].

Our preliminary results pointed to the possibility that lung cancer cells that ectopically express Vav1 (H358) also secrete CSF1, a hematopoietic growth factor that stimulates monocyte proliferation and differentiation into macrophages and can activate Vav1 in immune cells [21]. In inflammation, CSF1 induces macrophages to secrete cytokines and proteases, enhancing their ability to combat microbial infections [22]. Like other members of the growth factor receptor family, CSF1 receptor (CSF1R) regulates proliferation and differentiation of the monocyte lineage [22]. In lung cancer, CSF1 is one member of a three-gene signature strongly associated with poor prognosis in early-stage squamous cell carcinoma and its level of expression significantly increases with disease progression in Non Small Cell Lung Carcinoma (NSCLC) patients [23]. While increased CSF1 levels can be associated with malignancy and metastasis [24-27], tumor progression and development may also be dependent on whether CSF1 is derived from tumor cells, stromal cells or both [26].

Our results indicate that a CSF1-Vav1 pathway may contribute to lung cancer development. This intricate signaling pathways in which Vav1 and CSF1 are involved results on one hand in control of CSF1 expression in lung cancer cells and on the other hand, Vav1 stimulation by CSF1, thus evoking cytoskeleton organization and increased tumorigenicity. Furthermore, CSF1 secretion can affect the microenvironment of the tumor. Our studies also provide evidence for the first time for the involvement of Vav1 in a Rac-independent pathway in cancer cells.

## RESULTS

### Vav1 affects growth factor secretion

**Figure 1:**
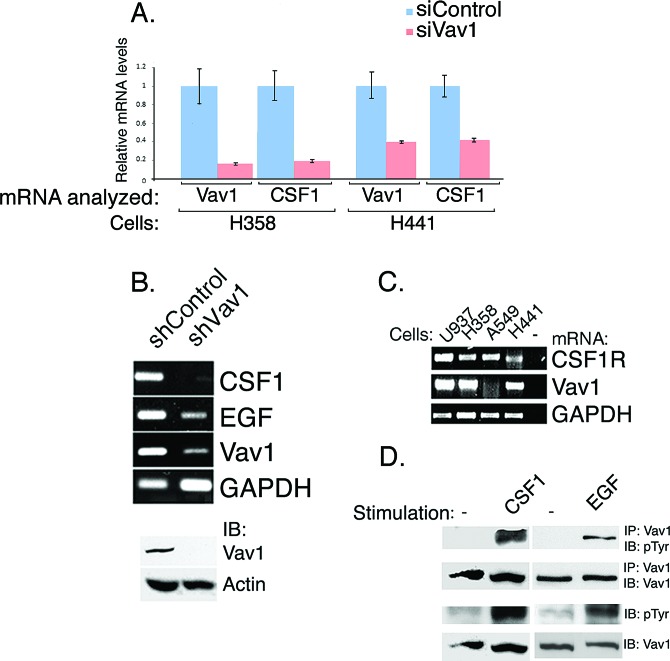
Vav1 affects CSF1 transcript expression while CSF1 affects Vav1 phosphorylation Vav1 was depleted in H358 and H441 cells by using siRNA, (A) and in H358 cells by shRNA (B). CSF1 and Vav1 mRNA levels were measured both by Real time PCR (A) and PCR (B). Cells treated with siControl are labeled with light blue, while cells treated with siVav1 are labeled in pink. The mRNA analyzed (Vav1 or CSF1) and the cells used (H358 or H441) are indicated. The levels of mRNA levels were calculated compared to that of the mRNA in siControl treated cells. The level of EGF mRNA expression was also measured (B). Depletion of Vav1 was also validated by immunoblottins (1B) (B, lower panel). (C) The level of CSF1R mRNA expression was tested in H358, H441 and A549 lung cancer cells, as well as in U937, a human leukemic monocyte lymphoma cell line (as indicated) by PCR. (D) H358 lung cancer cells were starved for 48hrs and were either non-stimulated (−) or stimulated 50 ng/ml human CSF1 or 100 ng/ml human EGF for 5 minutes (indicated). Cell lysates and cell lysates immunoprecipitated (IP) with anti-Vav1 polyclonal antibodies were resolved on SDS–PAGE and then immunoblotted (IB) with either anti-phosphotyrosine (pTyr) or anti-Vav1 monoclonal antibodies as indicated.

Our previous experiments indicated that lung cancer cells, H358, which ectopically express Vav1 can be grown in medium lacking growth factors for several days, thus suggesting that these cells secrete growth factors that support their growth [10]. Also, we demonstrated that Vav1-depleted H358 lung cancer cells exhibit a 40% reduction in the expression of TGFα, a growth factor that stimulates the Epidermal Growth Factor Receptor (EGFR; [10]) To further expand our knowledge on whether and how Vav1 might be involved in regulating cytokine secretion or other signaling pathways, we performed transcriptome analysis of H358 lung cancer cells in which Vav1 was depleted by siRNA and compared the level of down and up-regulated genes to their expression in control H358 cells (Tables 1 & 2, respectively). Table 1 presents a list of genes that exhibit at least 1.7-fold reduction in Vav1-depleted H358 cells, with a p-value of less than 0.07. The reduction in Vav1 mRNA expression is the greatest among the down-regulated genes, as expected. Among the genes that were down-regulated in the absence of Vav1 was CSF1 [21], as well as several genes whose products might participate in signaling events, including lysyl oxidase (LOX), known to participate in cancer development [28]. A comprehensive list is presented in Table S1. The genes which are up-regulated in Vav-depleted H358 cells include a variety of genes that participate in either signaling, cell cycle or serve as transcription factors (Table 2 & Table S2). The marked reduction in CSF1 mRNA in Vav1-depleted H358 cells (Table 1), was further substantiated by quantitative PCR performed on Vav1 siRNA transfected H358 cells (Figure 1A). Moreover these results were reproduced also in an additional lung cancer cell line, H441 (Figure 1A). Similar results were obtained when H358 were depleted of Vav1 using shRNA (Figure 1B). EGF mRNA expression was also reduced in Vav1-depleted H358 cells (Figure 1B), though its reduction was not as prominent as that of CSF1. Moreover, it was not included among the genes that were down-regulated in our transcriptome analysis (Table 1). Such a difference might stem from probable dissimilarities in the sensitivity of the various methods used or alternatively from the different cells used for the transcriptome [(depletion by siRNA (Table 1) versus the PCR experiment [depletion by shRNA (Figure 1B)]. As these results suggest that CSF1 may be a major mediator of a Vav1 dependent loop, we next wished to determine whether CSF1 leads to Vav1 tyrosine phosphorylation in lung cancer cells. Several reports indicated that Vav1 tyrosine phosphorylation is triggered by CSF1 in hematopoietic cells [21, 29]. However, there are no reports of Vav1 stimulation by CSF1 in cancer cells. To investigate this possibility, we explored the signaling events triggered by CSF1 stimulation in lung cancer cells. First, we confirmed the expression of CSF1R on H358 lung cancer cells (Figure 1C). Then, we stimulated H358 lung cancer cells, known to express high levels of Vav1 ([10]; Figure 1D, left panel), with CSF1. This induced significant tyrosine phosphorylation of Vav1. Similarly, Vav1 tyrosine phosphorylation was also observed following stimulation with EGF (Figure 1D, right panel). Thus, Vav1 affects CSF1 and other growth factor secretion and it is reciprocally activated by CSF1 stimulation. Taken together, our results suggest that Vav1 may propagate a putative autocrine feed forward loop by upregulating expression of CSF1, which in turn induces Vav1 tyrosine phosphorylation in cancer cells.

**Table 1:** List of down-regulated genes in Vav1 depleted H358 cells compared to control treated cells

Transcript ID	Fold-Change (SI vs. SC)	p-value (SI vs. SC)	Gene Function
VAV1	−4.52	0.00	Guanine Nucleotide Exchange Factor
LOX	−2.98	0.03	Lysyl Oxidase
CSF1	−2.11	0.02	Colony Stimulating Factor 1 (Macrophage)
RAB31	−1.97	0.07	Ras-Related Protein Rab-31
LOC338799	−1.80	0.04	lncRNA
STARD3NL	−1.80	0.05	STARD3 N-Terminal Like
TXLNG	−1.77	0.05	Taxilin Gamma
DIRC2	−1.77	0.03	Disrupted In Renal Carcinoma 2
PPAPDC2	−1.73	0.02	Phosphatidic Acid Phosphatase Type 2 Domain Containing 2
SMEK2	−1.71	0.06	SMEK Homolog 2, Suppressor Of Mek1

**Table 2:** List of up-regulated genes in Vav1 depleted H358 cells compared to control treated cells

Transcript ID	Fold-Change (SI vs. SC)	p-value (SI vs. SC)	Gene function
GNG5	3.77	0.00	Guanine nucleotide binding protein (G Protein), gamma 5
ITGA2	3.13	0.01	Integrin, Alpha 2 subunit of VLA-2 Receptor)
EREG	2.77	0.04	Epiregulin
ATXN10	2.20	0.06	Ataxin 10
ZDHHC6	2.19	0.05	Zinc Finger, DHHC-Type Containing 6
MNS1	2.17	0.03	Meiosis-Specific Nuclear Structural 1
ORC3L	2.17	0.01	Origin Recognition Complex, Subunit 3
ZNF45	2.16	0.00	Zinc Finger Protein 45
CKS2	2.10	0.02	CDC28 Protein Kinase Regulatory Subunit 2
RB1	2.10	0.07	Retinoblastoma 1
ZNF730	2.07	0.01	Zinc Finger Protein 730
PRDM1	2.07	0.05	PR Domain Containing 1, With ZNF Domain
KIAA0831	2.06	0.00	Autophagy Related 14
UGCG	2.05	0.05	UDP-Glucose Ceramide Glucosyltransferase
ZNF92	2.04	0.02	Zinc Finger Protein 92
PLEKHB2	2.01	0.01	Pleckstrin Homology Domain Containing, Family B
FAM73A	2.00	0.00	Family With Sequence Similarity 73, Member A
TNFRSF10A	2.00	0.02	Tumor Necrosis Factor Receptor Superfamily, Member 10a

### CSF1 affects signaling pathways in lung cancer cells

**Figure 2:**
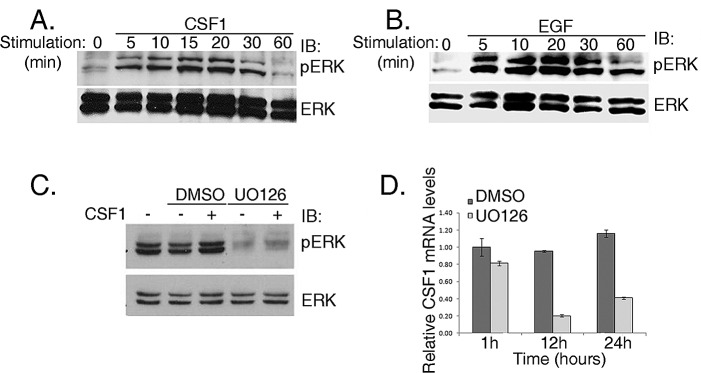
ERK phosphorylation via CSF1 stimulation in H358 cells is Vav1 dependent (A and B) H358 cells were starved for 48hrs and then stimulated with either CSF1 (A) or EGF (B) for various time intervals as indicated. Cell lysates were separated on SDS-PAGE and immunoblotted with either anti-pERK or anti-ERK antibodies, as indicated. (C) H358 cells were starved with serum-free medium for 48 hrs and then treated with 10 nM U0126 (MEK1 and MEK2 inhibitor) for an hour at 37^o^C. The cells were stimulated with CSF1 (+) or left untreated (-) for 5 minutes. Cell lysates were separated on SDS-PAGE and immunoblotted with anti-pERK or anti-ERK antibodies, as indicated. (D) H358 cells were treated with 10 nM U0126 (MEK1 and MEK2 inhibitor) as indicated in C. After an hour the cells were washed and fresh medium was added for various time points as indicated. CSF1 mRNA levels were measured by real time PCR.

Stimulation by CSF1 (Figure 2A) or by EGF (Figure 2B) led to transient ERK phosphorylation in H358 cells, first detected five minutes after stimulation and lasting at least 20 minutes. These results point to a signaling cascade that involves CSF1, Vav1 and ERK phosphorylation. To test whether the decrease in CSF1 transcription occurs via the ERK signaling cascade, we used the inhibitor of MEK1 and MEK2, U0126. U0126 inhibited CSF1-induced ERK phosphorylation in H358 cells (Figure 2C) and resulted in a significant decrease in CSF1 mRNA expression twelve and twenty-four hours later (Figure 2D). Thus, CSF1 expression in H358 cells is dependent on signal transduction pathways that involve Vav1 and ERK.

**Figure 3:**
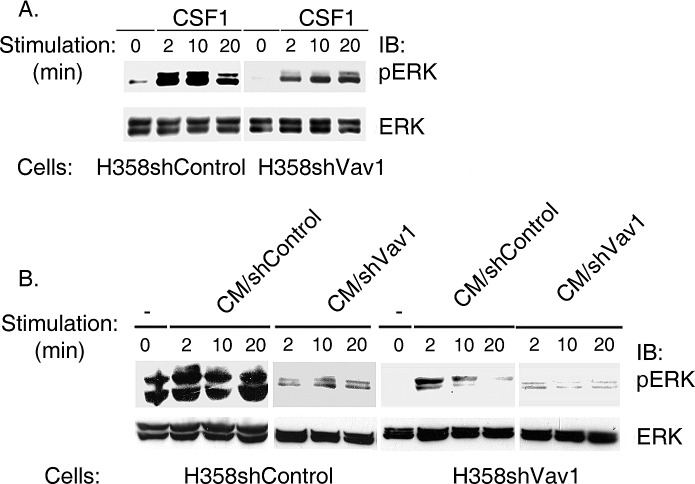
ERK phosphorylation is diminished following stimulation with conditioned media of Vav1-depleted H358 cells (CM/H358shVav1) (A) H358 infected with shVav1 (H358shVav1) or control (H358 shControl) were stimulated with CSF1 for various time points as indicated. Cell lysates were separated on SDS-PAGE and immunoblotted with anti-pERK or anti-ERK antibodies as indicated. (B) Conditioned media (CM) from H358 shVav1 (CM/ShVav1) or control (CM/shControl) prepared following stimulation of the cells with CSF1 as detailed in the Materials & Methods section was used to stimulate H358-shVav1 or shControl cells at various time points, as indicated. Cell lysates were separated on SDS-PAGE and immunoblotted with anti-pERK or anti-ERK antibodies, as indicated.

We next asked whether ERK phosphorylation via CSF1 stimulation in H358 cells is Vav1 dependent. We stably silenced Vav1 in H358 cells using shRNA (Figure 1B; H358shVav1), treated with CSF1, and assessed ERK phosphorylation. ERK phosphorylation was significantly reduced in H358-shVav1 cells compared to control cells (H358shControl), indicating an association between Vav1 and ERK phosphorylation in the CSF1 signaling pathway (Figure 3A). To determine whether CSF1 affects ERK phosphorylation via Vav1 expression, we stimulated starved H358shControl cells and H358shVav1 with conditioned medium (CM) collected from these cells following treatment for 10 minutes with CSF1 (Figure 3B). Stimulation of H358shVav1 cells with CM collected from control H358 cells (CM/shControl) showed considerable reduced but still detectable ERK phosphorylation compared to the effect of the same CM on H358shControl cells, suggesting that despite the presence of growth factors in this medium, H358shVav1 are defective in their response (Figure 3B, left panel vs. Figure 3B, right panel). Quantification of the results presented in Figure 3B are depicted in Supplementary Figure 1. Furthermore, there was no ERK phosphorylation in H358shControl and H358shVav1 cells following stimulation with CM/shVav1, demonstrating the requirement for Vav1 expression for growth factor secretion as well as signaling responses following CSF1 stimulation.

**Figure 4:**
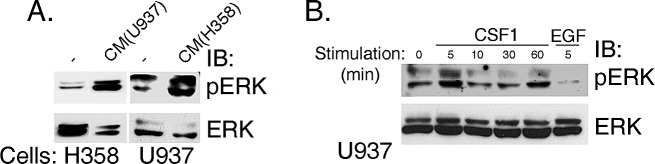
ERK is phosphorylated in H358 cells in response to stimulation with CM of U937, human leukemic monocyte lymphoma cell line cells and vice versa (A) Conditioned media was made by stimulating U937 and H358 cells with human CSF1 for 15 min. Cells were then washed to remove growth factors and fresh media was added for 48hr, after which the conditioned media were collected [CM (U937) and CM (H358), respectively]. U937 cells were treated with CM (H358) and vice versa. Cell lysates were separated on SDS-PAGE and immunoblotted with anti-pERK or anti-ERK antibodies, as indicated. (B) U937 cells were stimulated with CSF1 for various time intervals and with EGF for 5 minutes, as indicated. Cell lysates were separated on SDS-PAGE and immunoblotted with anti-pERK or anti-ERK antibodies, as indicated.

We then wondered what are the possible additional sources for CSF1 in the tumor microenvironment and hypothesized that tumor associated macrophages (TAMs) are a likely candidate. To analyze this possibility *in vitro*, we examined whether ERK is phosphorylated in H358 cells in response to stimulation with conditioned medium (CM) from human leukemic monocyte lymphoma-derived U937 cells [CM (U937)] (Figure 4A). As expected, ERK was phosphorylated under stimulation with CM (U937) (Figure 4A, left panel). U937 cells are known for secreting a large number of cytokines and chemokines [30]. Importantly, the reciprocal was also true: treatment with CM collected from H358 cells [CM/H358)] led to a marked increase in ERK phosphorylation in U937 cells (Figure 4A, right panel), indicating significant growth factor secretion by H358 cells. Thus, secreted factors from both cell types can activate the reciprocal cell type. Furthermore, U937 cells, which express CSF1R and Vav1, exhibited ERK phosphorylation following stimulation with CSF1, but not EGF (Figure 4B), thus indicating that U937 are responsive to CSF1, probably present in the medium of H358 cells. Taken together, the results thus far raise the possibility that Vav1 in lung cancer cells instigates a positive feed forward loop, which is amplified in the presence of macrophages: Vav1 stimulation induces cytokine secretion, which in turn activate macrophages to upregulate Vav1 expression (and possibly other cytokines such as EGF as well), resulting in increased ERK activation.

### Tumorigenic properties of Vav1 and CSF1 depleted lung cancer cells

**Figure 5:**
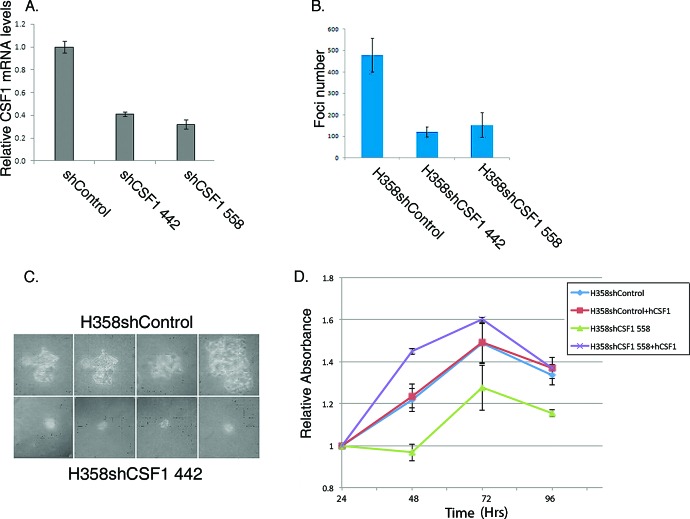
Depletion of CSF1 affects the tumorigenic properties of H358 lung cancer cells (A) Depletion of CSF1 reduces its mRNA expression. CSF1 was depleted in H358 cells using two different shRNA sequences against CSF1 (shSCF1 442 or shSCF1 558) or control (shControl; pLKO). The silencing efficiency of CSF1 was measured by real time PCR using specific primers. Actin was used as a control. (B) Depletion of CSF1 reduces focus formation. H358 cells infected with shRNA against CSF1 (H358shCSF1 442 and H358shCSF1 558) or control (H358shControl) were suspended in RPMI medium containing 0.3% agar and 10% calf serum, and plated onto a bottom layer containing 0.8% agar in a soft agar colony formation assay. Histograms show the mean of three independent experiments, each performed in triplicate ± SE (upper panel). The differences between the shControl and the shCSF 442 or shCSF 558 treatments were highly significant (*p* = 0.001 and 0.014 respectively; unpaired Student's *t*-test). (C) Photomicrographs of 14 days representative foci for each treatment are presented. (D) CSF1-depleted H358 cells are defective in their proliferation rate. H358shCSF1 558 or H358shControl cells were grown to sub confluence, and cells were either starved or supplemented with 50ng/ml hCSF1 for 24, 48, 72 or 96 hrs, as indicated, at which point proliferation rate was assessed by MTT incorporation. All conditions were performed in triplicate. Absorbance was quantified at 565 nm and calculated relative to the absorbance of each experimental point at its value at 24hrs (relative absorbance). Bars indicate Standard Errors.

We previously showed that Vav1 is important in lung tumorigenesis [10]. Our finding that Vav1 affects CSF1 expression and secretion and that the tumor microenvironment might also be involved in such a process prompted us to ask whether CSF1 is critical for Vav1 dependent lung tumor growth *in vitro*. We infected H358 cells with shRNA directed against CSF1 (Figure 5A). The CSF1-depleted cells exhibited greatly reduced ability to grow in soft agar compared with cells infected with scrambled DNA (Figure 5B). Moreover, the foci generated by these cells were considerably smaller than those in control-infected cells (Figure 5C). Both H358 shCSF1-infected cells also exhibited a significantly lower proliferation rate than control cells, as indicated by MTT assay (Figure 5D and data not shown). Convincingly, H358shCSF1 558 cells gained their ability to proliferate in a similar fashion to H358shControl cells when grown in medium supplemented with human CSF1 (hCSF1), thus proving that their growth defect stemmed from the knockdown of CSF1 and not from off target effects.

**Figure 6:**
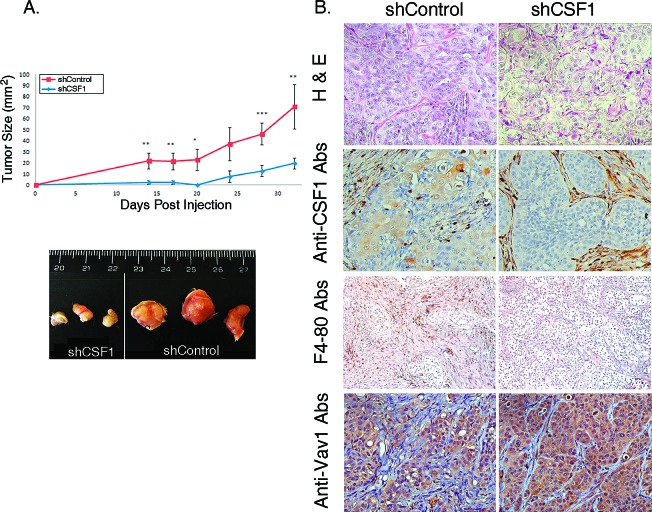
CSF1-depleted H358 cells have a lower rate of tumor growth in NOD/SCID mice (A) Rate of shCSF1 H358 cells tumor growth in vivo. 2×10^6^ H358 shCSF1 or shControl cells were injected subcutaneously into NOD/SCID mice. The growth rate of the resulting tumors was monitored and the tumor size (width × length) was calculated. (upper panel) Data points represent the mean of three experiments ± SE. The difference between the two groups was highly significant (*u*npaired Student's *t*-test * P=0.04, ** P=0.03, *** P=0.01). Photographs of representative tumors 33 days post-injection are shown (bottom panel). (B) Histological differences between shCSF1 and control tumors were shown by H&E staining, and immunohistochemistry of CSF1, F4-80 and Vav1, as indicated.

To examine the effect of CSF1 depletion on lung cancer cell tumorigenicity in an *in vivo* model, we injected H358 cells treated with either scrambled shRNA (shControl) or with shCSF1 vector (shCSF1) subcutaneously into the flank of athymic NOD/SCID mice and followed the appearance and growth rate of the injected cancer cells. shCSF1-treated H358 cells exhibited markedly reduced tumor growth rate (Figure 6A, upper panel) and final tumor size *in vivo* (Figure 6A, lower panel), compared with cells treated with shControl. shCSF1 tumors were also histologically different than control tumors, appearing more organized and fibrotic, as shown by H&E staining (Figure 6B, H&E), and with markedly reduced macrophage infiltration, as shown by F4-80 staining (Figure 6B, F4-80). Thus, expression of CSF1 is critical for the tumorigenic properties of H358 lung cancer cells.

### CSF1 and Vav1 are expressed in primary human lung cancer

**Figure 7:**
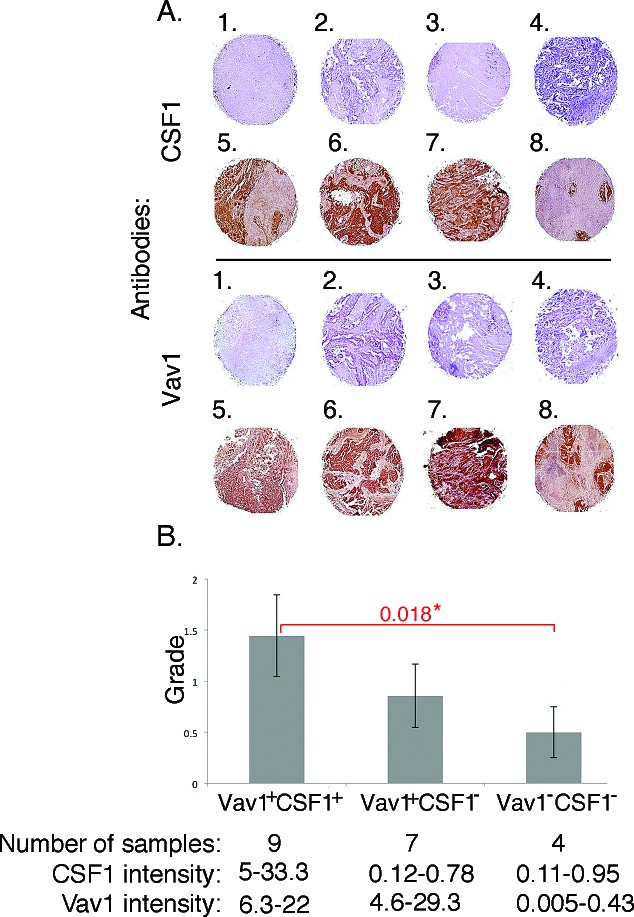
Correlation between CSF1 and Vav1 expression in human lung tumors (A) Paraffin-embedded sections of human lung cancer were incubated with either anti-Vav1 polyclonal antibodies or with anti-CSF1 antibodies. In total, 57 spots with diameter 1.5 mm were analyzed. Several lung tumor specimens either negative for both Vav1 and CSF1 (1-4) or positive for both Vav1 and CSF1 (5-8) are presented. (B) The correlation between Vav1^+^/CSF1^+^, Vav1^+^/CSF1^−^, and Vav1^−^/CSF1^−^ and tumor grade is depicted. The intensity of tumor staining was determined as detailed in the Material and Methods section. The number of samples and the intensity of Vav1 and CSF1 staining in each group are outlined at the bottom of this figure. Also, the statistical significance between the Vav1^+^/CSF1^+^ compared to Vav1^−^/CSF1^−^ groups (0.018) is indicated.

We previously reported Vav1 expression in 26/57 (45%) malignant lung samples, including adenocarcinoma, squamous cell carcinoma and adenocarcinoma with lepidic growth [10]. Immunostaining of the same samples for CSF1 revealed its expression in 42% of the same specimens. Staining intensity was assessed using an automated robotic image analysis system. Using this objective measure, 23% specimens were considered not stained (intensity score *<*1), 35% had low-intensity cytoplasmic staining (1–4), 27% had moderate cytoplasmic staining (5–10) and 15% were highly stained in the cytoplasm (*>*10) (Table S4). Remarkably, we found a significant positive correlation between expression of Vav1 and CSF1 in these primary human lung cancer specimens (p<0.05). Figure 7A shows some examples of lung tumor specimens that are either negative for both Vav1 and CSF1 (1-4) or positive for both Vav1 and CSF1 (5-8). While there was no significant correlation between tumor grade and either Vav1 or CSF1 expression alone, the expression of both Vav1 and CSF1 together was positively correlated with higher tumor grade. Lack of both Vav1 and CSF1 correlated with lower tumor grade (p<0.05; Figure 7B). Interestingly, we also identified a group of tumors where CSF1 is low while Vav1 is highly expressed (Figure 7B).

Thus, combined Vav1 and CSF1 expression correlates with tumor grade in human lung cancer samples, providing support for the hypothesis that Vav1 and CSF1 might have converging roles in lung cancer development.

## DISCUSSION

The contribution of Vav1 to human cancer has been shown to stem from its ability to function as a signal transducer in tissues in which it is not normally expressed [3]. Vav proteins are tyrosine phosphorylated *in vitro* by Syk [31], Lyn [32] and Fyn [33] and in response to EGF and PDGF stimulation in NIH3T3 fibroblasts [12, 13] and pancreatic [9] and lung cancer [10]. Our current studies demonstrate for the first time, that Vav1 is tyrosine phosphorylated in response to CSF1 in lung cancer cells, thus suggesting a supportive role for Vav1 as a universal signal transducer in cancer. Tyrosine phosphorylation of Vav1 following stimulation of various receptors leads to its activation, which triggers downstream signaling cascades. Indeed, depletion of Vav1 in lung cancer cells led to reduced ERK phosphorylation despite stimulation with CSF1. Numerous studies have implicated Vav1 in ERK [34-36], JNK [37] and PLCγ phosphorylation [38], but most of these studies were done in hematopoietic cells, where Vav1 is physiologically active. Our studies clearly show that ectopically expressed Vav1 plays a role in ERK phosphorylation in non-hematopoietic cells as well.

Thus far, the main role attributed to Vav1 in cancer was its regulation of the activity of Rho/Rac GTPases. These proteins function as molecular switches in a variety of signaling pathways following stimulation of cell surface receptors [15]. For instance, Rho/RacGTPases regulate numerous cellular processes including cytoskeleton organization, gene transcription, cell proliferation, migration, growth and survival [39]. Because of their central role in regulating processes that are dysregulated in cancer, it seems reasonable that defects in the RhoGTPase pathway may be involved in the development of cancer [40]. Indeed, Vav1-depletion in pancreatic and lung cancer cell lines results in the reduction of colony formation in soft agar *in vitro* and reduction of tumor size in immunocompromised mice [9, 10]. Interestingly, this influence of Vav1 expression was observed even in the presence of mutant K-Ras, demonstrating the critical role of Vav1 in tumor development [9,10].

One of the most intriguing results from our current studies is that lung cancer cells depleted of Vav1 exhibit significantly reduced levels of CSF1, suggesting that Vav1 propagates an autocrine feed forward loop by upregulating expression of growth factors. Thus, based on our results, Vav1 might be involved in additional pro-tumorigenic pathways as well as its GEF activity.

The possibility that Vav1 can stimulate secretion of autocrine ligands was also suggested for the human mammary epithelial cell line MCF-10A, in which expression of a constitutively active form of Vav1 promoted migration and morphological changes [41]. This increased migration was dependent on Vav1 GEF activity, which stimulated the Rac1–Pak pathway, and also on secretion of an autocrine EGF receptor ligand. Similar to our results with CSF1, in lung cancer cell lines, TGFα leads to an increase in tyrosine phosphorylation of Vav1, while depletion of Vav1 reduces TGFα expression [10]. Interestingly, even the expression of EGF is reduced in Vav1-depleted cells. These data support the existence of feed-forward loops in which Vav1 regulates secretion of autocrine ligands leading to receptor stimulation and subsequent increases in Vav1 activation. The expression and function of many other proteins appear to be affected by Vav1 (Tables 1 & 2). We previously reported that the secretion of osteopontin, a CD44 and integrin ligand known to be associated with invasion, progression and metastasis, is upregulated by oncogenic Vav1 in NIH3T3 cells [42]. Fernandez-Zapico *et al*., demonstartated that the expression of Vav1 in pancreatic cancer involves cyclin D1 upregulation [9]. This recurring theme suggests that Vav1 might contribute to the progression of cancer by regulating secretion of autocrine ligands critical for tumorigenicity, as well as affecting the expression of other proteins critical for various functions in the cell, as noted by us herein for the first time (Tables 1 & 2). Further studies will be needed to be executed to clarify the function and significance of these pathways for cancer.

CSF1-depleted lung cancer cells demonstrate reductions in proliferation and focus-formation *in vitro* and reduced tumor growth in immune-compromised mice, indicating that CSF1 expression and secretion is cardinal for tumorigenicity. Indeed, the contribution of CSF1 to transformation was previously demonstrated when NIH 3T3 cells were coinfected with the human c-fms proto-oncogene together with CSF-1 underwent transformation by an autocrine mechanism [43]. Furthermore, a previous report provided a direct *in vivo* evidence that similar autocrine mechanisms function in H358 cells, for instance HGF-Met signaling plays significant roles in the growth and differentiation of these cells [43].

Our study reveals that Vav1 and CSF1 influence the activity of each other and are engaged in an autocrine mechanism that could enhance tumor growth. This secretory mechanism also influences the tumor microenvironment. Our results support this possibility, showing that CM from H358 lung cancer cells leads to increased signaling in the monocytic cell line U937 and vice versa. Also, tumors of H358 cells developed in immune-compromised mice exhibit increased macrophages in the vicinity of the tumor compared to tumors of CSF-depleted H358 cells. The importance of CSF1 to tumor development and to the stromal cells has been recognized recently in various experimental systems [44-49]. The importance of CSF1 was further highlighted by the demonstration that metastasis from the mammary gland to the lungs was significantly attenuated in CSF1 knock-out mice [29, 47, 48]. This correlated with a reduction in the number of macrophages recruited to the mammary tumors. Similarly, mammary-specific overexpression of CSF1 accelerated the progression to malignancy and enhanced metastasis in MMTV-PyMT mice with normal systemic levels of CSF1 [50]. The use of anti-sense and siRNA toward CSF1 or its receptor further demonstrated its role in growth of breast cancer xenografts [44]. Administration of CSF1 to mice inoculated with neuroblastoma cells led to an increase in tumor growth followed by an increase in systemic levels of VEGF and increases in tumor angiogenesis [49]. For instance, a reciprocal interaction exists between macrophages and breast cancer cells whereby the tumor cells produce CSF1 and express the receptor for EGF while macrophages produce EGF and express the receptor for CSF1 [45, 46].

Finally, the correlation we observed between combined Vav1 positive/CSF1 positive expression and tumor grade further strengthens our conclusion that these proteins act together in a feed-forward loop that contributes to tumorigenicity in human lung cancer.

## MATERIALS AND METHODS

### Cell culture, cell stimulation and inhibition

H358 (bronchioalveolar non-small lung carcinoma), H441 (Lung Papillary Adenocarcinoma) and A549 (Lung Carcinoma) kindly given to us by Drs. Gazdar and Minna [51], as well as U937 (monocytes, histiocytic lymphoma [52]) were grown in RPMI medium (Sigma). All media was supplemented with 10% Fetal Bovine Serum (FBS), Penicillin-Streptomycin and L-Glutamine (Biological Industries, Israel) and cells were maintained at 37°C with 5% CO_2_. For stimulation with CSF1 or EGF, cells were grown to sub-confluence, starved in serum-free medium for 48hr and treated with 50 ng/ml human CSF1 (Peprotech, NJ, USA) or 100 ng/ml human EGF (Cytolab, Rehovot, Israel) for various time points as indicated. For conditioned media (CM) experiments, the indicated cells were stimulated with 50 ng/ml human CSF1 (Peprotech, NJ, USA) for 10 min or 100 ng/ml human EGF for 5 min. Cells were then washed to remove growth factors and fresh medium was added. 48hr later, the conditioned medium was collected. For U2106 treatment, H358 cells were starved with serum-free medium for 48hr and then treated with 10 nM U0126 (MEK1 and MEK2 inhibitor; Cell Signaling Technology, MA, USA) for one hour at 37 degrees. Cells were then washed, fresh media added for various time points, as indicated.

### DNA microarrays

Gene expression profiling was performed using the Affymetrix Human Gene 1.0 ST Array (Santa Clara, CA), according to the manufacturer's instructions. Expression of genes in Vav1-depleted by siRNA H358 compared to H358 cells transfected with scrambled siRNA, as previously described [10], was performed. The results of two independent experiments were averaged. Genes that were up- or down-regulated by at least 1.5 fold with a *P-*value of ≤ 0.07 were considered statistically significant.

### Antibodies

Cell lysis, immunoprecipitation (IP), and immunoblotting (IB) procedures were performed as described [10] using the antibodies outlined in Table S3. Immunostaining was performed as described below using the antibodies outlined in Table S3.

### Immunohistochemistry of Human Lung Tissue Array

Human 1ung paraffin tissue array (http://www.biochain.com/biochain/datasheet/Z7020004-B410017.pdf) was purchased (Biochain, CA, USA) and treated according to manufacturer's instructions. Immunostaining was performed using the labeled streptavidin biotin (LAB-SA) technique (Histostainplus, Cat. No. 85–8943, Zymed Laboratories, CA, USA) on 5 μM sections according to the manufacturer's instructions. Staining was evaluated by a board certified pathologist (EP). Staining intensity was quantified using an Ariol SL-50 image analysis system (Applied Imaging, UK), using the TMAsight assay. In each tissue core on the array, a pathologist identified neoplastic foci and the intensity of brown staining was evaluated. The calculated mean intensity index represents summed positive pixel intensities divided by the area of the detected foci. In total, 57 spots with diameter 1.5 mm were analyzed (52 were confirmed as cancer specimen by EP.). The mean analyzed area (e.g. area of tumors selected for analysis) for each core was 0.15 mm^2^.

### RT-PCR

Total RNA and reverse transcription of Vav1, CSF1, CSF1R, EGF and GAPDH was performed as previously described [10] (Table S5).

### Quantitative Real-time PCR

Total RNA and cDNAs from cell lines were prepared as for RT-PCR [10]. Detection of Vav1, CSF1 and was performed using cyber green PCR master mix (Tamar, Jerusalem, Israel) and the required primers (Table S5). Analysis was performed using the ABI Prism 7300 real-time PCR technology (Applied Biosystems, CA, USA). Three independent experiments were performed, each in triplicate.

### MTT Cell Proliferation Assay

H358shControl and H358shCSF1 558 cells were grown in 12 well plates to sub-confluence and then either starved for the length of the experiment (96 hours) or supplemented every 24hrs with 50 ng/ml human CSF1 (+hCSF1)(Peprotech, NJ, USA) or with vehicle control (water and 0.1% BSA used to dilute CSF1 according to the manufacturer's instructions; Peprotech, NJ, USA). To assess proliferation, 0.1 mg/ml of MTT [3-(4,5-Dimethylthiazol-2-yl)-2,5-diphenyltetrazoliumbromide] in dimethyl sulfoxide was added to wells of each cell type at 24hrs, 48hrs, 72hrs and 96hrs. All conditions were performed in triplicate. Absorbance was quantified at 565 nm.

### Soft Agar Colony Formation Assay

The soft agar assay was carried out as previously described [10]. Three independent experiments were performed, each one in triplicate.

### Silencing gene expression by shRNA

Cells were infected with pLKO-based (Open Biosystems) lentiviral vector with or without the human CSF1, Vav1- shRNA encoding sequences (Table S5). Infected cells were selected with puromycin.

### Tumorigenicity assay using NOD/SCID mice

We injected 2×10^6^ H358 lung cancer cells in 100 μl and an equal volume of Matrigel (BD Biosciences) subcutaneously into NOD/SCID mice and measured tumor growth twice weekly. Tumor size was calculated as the multiplication of the width and length (mm^2^). Mice were sacrificed 33 days post-injection and tumor cuts were subjected to immunohistochemistry using anti-Vav1, anti-CSF1 and anti-F4-80 according to the manufacturer's instructions. Each experimental group contained six mice and the experiments were performed three times. Staining was evaluated by a board certified pathologist (E.P). The Animal Facility at the Hebrew University, following NIH guidelines, approved the procedures used for these experiments.

## SUPPLEMENTARY MATERIAL, TABLES AND FIGURES






