# Supplementary material for: Vav1 promotes lung cancer growth by instigating tumor-microenvironment cross-talk via growth factor secretion
Source: Oncotarget. 2014 Aug 27;5(19):9214–26. doi: 10.18632/oncotarget.2400 (PMC4253429; doi:10.18632/oncotarget.2400)
Supplement: Supplementary file 1 [file oncotarget-05-9214-s001.pdf]

Vav1 promotes lung cancer growth by instigating tumor-microenvironment cross-talk via growth factor secretion

Supplementary Material

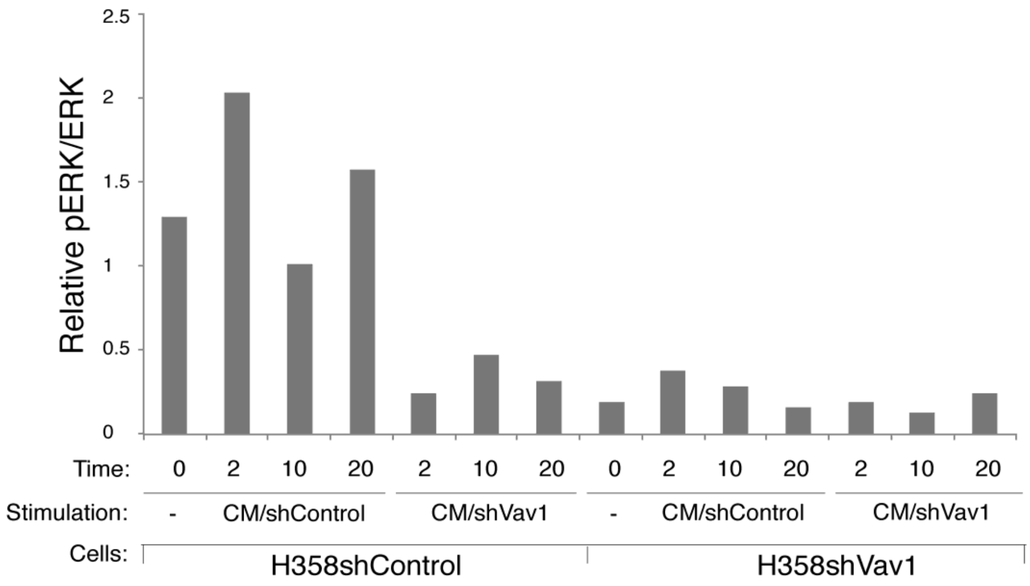

Supplementary Figure 1: Quantification of ERK phosphorylation following stimulation with conditioned media of Vav1-depleted H358 cells (CM/H358shVav1).

Table S1: Comprehensive list of down-regulated genes in Vav1 depleted H358 cells compared to control treated cells.

Transcript ID	Fold-Change (SI vs. SC)	p-value (SI vs. SC)
VAV1	-4.52	0.00
LOX	-2.98	0.03
CSF1	-2.11	0.02
RAB31	-1.97	0.07
LOC338799	-1.80	0.04
STARD3NL	-1.80	0.05
TXLNG	-1.77	0.05
DIRC2	-1.77	0.03
PPAPDC2	-1.73	0.02
SMEK2	-1.71	0.06
RETSAT	-1.69	0.01
SNX13	-1.68	0.06

MRGPRX3	-1.66	0.01
TRAK2	-1.66	0.05
SLC34A2	-1.65	0.01
JAK2	-1.65	0.00
FAM115A	-1.64	0.06
PCK2	-1.64	0.04
FAM82A2	-1.64	0.03
C6orf168	-1.63	0.04
LRRC16A	-1.61	0.04
LARP4B	-1.61	0.09
ZNF558	-1.60	0.07
BHLHA15	-1.59	0.05
PFN4	-1.58	0.00
BCAP31	-1.57	0.03
KLF12	-1.57	0.04
ACTR1B	-1.56	0.00
KCTD18	-1.56	0.01
MIR516B1	-1.56	0.01
CXXC4	-1.55	0.03
CKAP4	-1.54	0.02
C10orf46	-1.53	0.04
MON1B	-1.53	0.00
GALNT3	-1.53	0.03
MCOLN3	-1.52	0.00
DHX29	-1.52	0.06
NCAM2	-1.52	0.05
RAB33B	-1.52	0.04
ZNF385A	-1.51	0.02
IRF2	-1.51	0.02
TMED8	-1.51	0.03
MT1L	-1.51	0.02
RCAN2	-1.51	0.06
NUMB	-1.51	0.03
SCGBL	-1.50	0.07

Table S2: Comprehensive list of up-regulated genes in Vav1 depleted H358 cells compared to control treated cells.

Transcript ID	Fold-Change (SI vs. SC)	p-value (SI vs. SC)
GNG5	3.77	0.00
ITGA2	3.13	0.01
EREG	2.77	0.04
ATXN10	2.20	0.06
ZDHHC6	2.19	0.05
MNS1	2.17	0.03
ORC3L	2.17	0.01
ZNF45	2.16	0.00
CKS2	2.10	0.02
RB1	2.10	0.07
ZNF730	2.07	0.01
PRDM1	2.07	0.05
KIAA0831	2.06	0.00
UGCG	2.05	0.05
ZNF92	2.04	0.02
PLEKHB2	2.01	0.01
FAM73A	2.00	0.00
TNFRSF10A	2.00	0.02
MCFD2	1.99	0.02
BRMS1L	1.97	0.03
NMU	1.93	0.03
CDK1	1.92	0.05
ZNF695	1.89	0.00
CKAP2L	1.89	0.03
ZNF28	1.88	0.02
SNORD30	1.83	0.04
HSPA2	1.83	0.00
AGPAT9	1.82	0.07
CHCHD4	1.81	0.01
ZRANB3	1.81	0.01
FAM72D	1.80	0.05
HSPH1	1.80	0.07
RPL7L1	1.78	0.04
LCLAT1	1.76	0.01
RGS2	1.76	0.05
PRR11	1.75	0.04

NUFIP1	1.75	0.04
TMED5	1.75	0.03
NEK2	1.73	0.01
PRDX3	1.73	0.07
SGOL1	1.72	0.02
VPS4B	1.72	0.00
SPATS2L	1.71	0.03
G2E3	1.71	0.01
ZNF675	1.71	0.00
MRTO4	1.70	0.01
RNF138	1.70	0.04
C4orf21	1.70	0.07
TMEM66	1.69	0.05
NUSAP1	1.69	0.01
LRRC40	1.69	0.07
RSRC1	1.68	0.02
MTAP	1.67	0.01
HSPA4L	1.67	0.04
TFRC	1.66	0.02
C3orf23	1.66	0.04
FAM54A	1.66	0.07
PRPS1	1.64	0.05
HAUS6	1.64	0.01
ORC2L	1.64	0.04
PARP2	1.64	0.00
LOC643837	1.63	0.03
BRD8	1.63	0.05
SLC20A1	1.62	0.03
SENP2	1.62	0.02
C1orf116	1.61	0.07
LOC402644	1.60	0.01
RPA2	1.60	0.05
CCDC75	1.60	0.01
POLR3G	1.60	0.01
CCDC103	1.59	0.06
UTP23	1.59	0.03
RYBP	1.59	0.00
SLC38A9	1.59	0.07
VTA1	1.58	0.02
TIMM50	1.58	0.01
LTV1	1.58	0.07

RNF11	1.58	0.07
APOO	1.57	0.06
FAM76B	1.57	0.03
ITGB3BP	1.57	0.00
TAF9B	1.57	0.06
VSIG1	1.56	0.01
FBXO45	1.56	0.02
LOC647302	1.56	0.02
ELF2	1.55	0.01
PLRG1	1.55	0.01
TECR	1.55	0.05
GTF2B	1.55	0.03
ZNF121	1.55	0.03
NSUN4	1.55	0.07
CENPQ	1.55	0.06
TM2D2	1.54	0.06
ZNF702P	1.54	0.03
TDG	1.54	0.01
CCDC21	1.54	0.05
SLC25A19	1.54	0.07
INPP1	1.52	0.02
RPP40	1.52	0.01
RNF5P1	1.51	0.05
AMD1	1.51	0.07
TMEM126B	1.51	0.07
DCK	1.51	0.02
ZNF468	1.51	0.03
AURKA	1.51	0.07
MRPS18B	1.51	0.02
KPNA4	1.50	0.05
MND1	1.50	0.05
ZNF681	1.50	0.06

Table S3: Antibodies used for Immunoprecipitation, Immunoblotting and Immunohistochemistry.

Name of Antibodies & Purpose	Manufacturer information/Refernce
Monoclonal anti-Vav1	Upstate Biotechnology, NY, USA; #05-219
Anti-F4-80	AbD Serotec, NC, USA; #4976
Anti-phosphotyrosine	Millipore, MA, USA; #05-321
Anti-ERK	Millipore, MA, USA; #06-182
Anti- CSF1	ABGENT, USA #AJ1466a
Anti-pERK	Cell Signaling, MA, USA; #9106S
Anti-actin	Santa Cruz, USA; #SC130656
Rabbit polyclonal anti-Vav1 antibodies	Katzav S, et al. (1991) *Mol. Cell. Biol.* 11: 1912-1920
