# Supplementary material for: Vav1 promotes lung cancer growth by instigating tumor-microenvironment cross-talk via growth factor secretion
Source: Oncotarget. 2014 Aug 27;5(19):9214–26. doi: 10.18632/oncotarget.2400 (PMC4253429; doi:10.18632/oncotarget.2400)
Supplement: Supplementary file 3 [file oncotarget-05-9214-s003.docx]

Table S5: Primers used for PCR, Real-Time PCR and shRNA sequences.

| shRNA sequences | Reverse primer | Forward primer | Gene |
| --- | --- | --- | --- |
|  | 5'-GCTTGGTGTTGTTGTGTTGG-3' | 5'-ACAGGGCCAAGTTCATTCAG-3' | CSF1R |
|  | 5'-TCCAGCAACTGGAGAGGTGTCTCATA-3' | 5'- TGCAGGAACTCTCTTTGAGGCTGA-3' | CSF1 |
|  | 5'-TTCGTGAGCTCCACAATGTCTCCA-3' | 5'-TCTGCCCAAGATGGAGGTGTTTCA-3' | Vav1 |
|  | 5'-AAGCTTCGCTCCATTACCTG-3' | 5'-CAATGCAACCAACTTCATGG-3' | EGF1 |
|  | 5'-ACCAAATCCGTTGACTCCGACCTT-3' | 5'-TCGACAGTCAGCCGCATCTTCTTT-3' | GAPDH |
| CCGGCGTCGAGGTCAAGCACATTAACTCGAGTTAATGTGCTTGACCTCGACGTTTTTG |  |  | VAV1 |
| CCGGCCACTATTTATTGTGAGCCCTCTCGAGAGGGCTCACAATAAATAGTGGTTTTTG |  |  | CSF1 558 |
| CCGGTCTCCTGGTACAAGACATAATCTCGAGATTATGTCTTGTACCAGGAGATTTTTG |  |  | CSF1 442 |
